# An Efficient 3D Human Pose Retrieval and Reconstruction from 2D Image-Based Landmarks

**DOI:** 10.3390/s21072415

**Published:** 2021-04-01

**Authors:** Hashim Yasin, Björn Krüger

**Affiliations:** 1 School of Computing, National University of Computer and Emerging Sciences, Islamabad 44000, Pakistan; 2 Gokhale Method Institute, Stanford, CA 94305, USA; bjoern@gokhalemethod.com

**Keywords:** motion capture, feature sets, 3D human pose retrieval, knowledge-base, 3D articulated pose estimation, optimization

## Abstract

We propose an efficient and novel architecture for 3D articulated human pose retrieval and reconstruction from 2D landmarks extracted from a 2D synthetic image, an annotated 2D image, an *in-the-wild* real RGB image or even a hand-drawn sketch. Given 2D joint positions in a single image, we devise a data-driven framework to infer the corresponding 3D human pose. To this end, we first normalize 3D human poses from Motion Capture (MoCap) dataset by eliminating translation, orientation, and the skeleton size discrepancies from the poses and then build a *knowledge-base* by projecting a subset of joints of the normalized 3D poses onto 2D image-planes by fully exploiting a variety of virtual cameras. With this approach, we not only transform 3D pose space to the normalized 2D pose space but also resolve the 2D-3D cross-domain retrieval task efficiently. The proposed architecture searches for poses from a MoCap dataset that are near to a given 2D query pose in a definite feature space made up of specific joint sets. These retrieved poses are then used to construct a weak perspective camera and a final 3D posture under the camera model that minimizes the reconstruction error. To estimate unknown camera parameters, we introduce a nonlinear, two-fold method. We exploit the retrieved similar poses and the viewing directions at which the MoCap dataset was sampled to minimize the projection error. Finally, we evaluate our approach thoroughly on a large number of heterogeneous 2D examples generated synthetically, 2D images with ground-truth, a variety of real *in-the-wild* internet images, and a proof of concept using 2D hand-drawn sketches of human poses. We conduct a pool of experiments to perform a quantitative study on PARSE dataset. We also show that the proposed system yields competitive, convincing results in comparison to other state-of-the-art methods.

## 1. Introduction

Understanding of human motion and the analysis of human behavior have been widely studied and investigated by researchers from various domains in the last few decades. The estimation of human poses, either in the 2D or 3D domain, may be considered a key component in analyzing human behavior. Thus, the demand and need to capture and generate 3D human motion are continuously increasing [1]. There exist a variety of professional systems to capture human motions, i.e., magnetic or acoustic-based systems [2], optical motion capture systems [3], and virtual marker-based systems [4,5] like *Vicon*, *MX* and *Giant*, etc. Among all these MoCap systems, virtual marker-based MoCap systems are prevalent and considered conventional and standard techniques. However, these systems require an indoor studio-like hardware setup, which is highly expensive. In practice, the marker-attached suit and the studio-like indoor environment prevent capturing of realistic human motions in some cases. Additionally, these systems need extensive post-processing and after-efforts to deal with missing and corrupted data to generate accurate 3D human captured motions [6]. As an alternative, depth cameras (like Microsoft Kinect), which are less expensive, easy to handle, and more convenient in handling, have been used. The depth cameras hold a significantly less operational range, i.e., 1.0 m to 4.0 m. They are not very feasible in outdoor environment as the infrared system of the depth cameras is easily distorted by sunlight.

Despite all these above-mentioned MoCap systems, we have to deal with a bulk of *in-the-wild* 2D images/videos available on the internet and social media, which have no depth information at all. Hence, to meet the massive demand for MoCap data, many research works have been performed to infer 3D human poses from internet-based *in-the-wild* real 2D images/videos [7,8,9,10,11,12,13]. Due to the curse of dimensionality and the ill-posed nature [14], there are open challenges connected to lifting 2D poses up to 3D poses. 3D human motion capturing from *in-the-wild* 2D pictures and videos will empower many vision-dependent applications such as health rehabilitation-based industries, robotics, virtual reality, entertainment, surveillance systems, and human-computer interaction [15]. We propose a data-driven architecture that first searches for and retrieves K nearest neighbors (Knn) from MoCap datasets and then uses these retrieved Knn for online 3D articulated human pose configurations from the given input 2D image landmarks. The reconstruction of a 3D articulated human pose from a monocular, single, and static 2D image is an ill-posed problem as: (i) there may be multiple 3D human poses matching a single static 2D image-based pose, (ii) the completely unknown camera parameters, (iii) absolute missing of depth knowledge, (iv) the irreversible nature of the weak perspective projections. Our data-driven pipeline depends on efficient and fast searching and retrieval of a fixed-number of similar poses from the underlying motion capture dataset. Thus, we devise and investigate a variety of feature sets consisting of different joint combinations. In this way, we make the process of searching and retrieval more convenient and proficient. Once found, these Knn are utilized to estimate the unknown camera parameters and predict the final 3D articulated human pose. As input to the proposed system, we use 2D landmarks that are extracted from the (a) heterogeneous 2D synthetic examples created from MoCap data by utilizing some random camera parameters, (b) detected or annotated 2D pose in RGB images (c) *in-the-wild* real images, or (d) hand-drawn sketches of human postures. We provide 2D feature sets to the system drawn out from the single static 2D input query and then search for and retrieve Knn from the developed *knowledge-base*. The *knowledge-base* incorporates normalized 3D pose space and the relevant normalized 2D pose space established through an orthographic projection of normalized 3D pose utilizing various virtual cameras. We, in fact, develop the *knowledge-base* to address the issue of the 2D-3D correspondence. As a result, we efficiently deal with the 2D-3D cross model Knn search and retrieval. We benefit from these K nearest neighbors in several ways: (i) We first predict the unknown camera parameters utilizing these Knn combining with the information of the view directions at which MoCap data is sampled. (ii) We also learn a local pose model using these retrieved Knn in a Principal Component Analysis (PCA) space.

We optimize the low-dimensional local model through our objective function with different error terms, i.e., retrieved pose error and the projection error, using a gradient-descent-based optimization algorithm. We perform quantitative evaluations of our proposed architecture on a pool of synthetic 2D images, collected from a variety of activities. We work on benchmark MoCap datasets, i.e., CMU MoCap dataset [16], HDM05 MoCap dataset [17], and Human3.6M [18]; all these datasets are available publically. We also report the qualitative evaluations of our proposed approach on *in-the-wild* real images taken from PARSE dataset [19] and 2D hand-drawn human poses. We compare the proposed system with other state-of-the-art methods. The results yield that our architecture executes compelling performance comparatively.

We organize this work as follows: We first illustrate the existing 3D pose prediction approaches in Section 2. We demonstrate all the necessary steps of the proposed architecture in Section 3. In Section 4, we describe and discuss the experiments as well as the obtained results. A comparison of our methodology with other state-of-the-arts is also discussed in detail in Section 4. In the end, we conclude this work in Section 5.

## 2. Related Work

Human motion capturing and the analysis of the generated motion data is a rapidly growing area in computer vision, computer graphics, and human-computer interaction. A lot of research has already been done on 3D reconstruction and analysis of human motions or poses. Recently, the popularity of 3D human pose prediction from a single static 2D images are growing day by day. The literature for 3D human pose prediction may be categorized as (i) generative approaches [20,21,22], which depends on the best possible alignment with the image descriptors/features and focus on modeling the underlying patterns of the image descriptors/features. These approaches require some realistic and reasonable initializations. (ii) The discriminative models or conditional models [23,24,25,26,27,28] do not rely on the image features’ alignment. Still, they aim to find the decision boundary and ultimately direct 3D mapping from the input data. A few approaches [23,25,26,29,30] exploit discriminative techniques to learn a model from the image descriptors (e.g., HOG, SURF, SIFT, etc.) to estimate 3D articulated human pose. At the same time, some works use a deep CNN [27,28]. (iii) The deep learning-based approaches [12,14,27,28,31,32] which do not rely on hand-crafted features/descriptors but learn features and mapping to 3D human poses directly. (iv) There also exist hybrid approaches [6,33,34] that combine together the generative as well as discriminative methods. The authors in [33] estimate 3D human pose by fully exploiting the generative probabilistic kinematic model for the 3D human pose hypothesis and the discriminative 2D body part detectors that weigh those hypotheses. In [34], 3D Pictorial Structure Model (PSM) is proposed where the regression forests are learned to predict 3D joint location probabilities, and ultimately the PSM optimizes the 3D articulated pose.

There exist a bulk of approaches that predict 3D articulated pose in a semi-supervised fashion. Zhou et al. in [11] propose a transfer learning approach in a weakly-supervised way. They train their network in an end-to-end manner and predict 2D pose and learn to estimate the depth information simultaneously. Yang et al. in [9] propose a dual-source adversarial learning approach, where they introduce the multi-source discriminator that is learned to distinguish the estimated 3D poses from the ground-truth poses. As a result, the pose estimator is forced to produce plausible poses that are anthropometrically valid, even with the unannotated *in-the-wild* images. In [13], the authors propose a fully CNN architecture that deploys temporal convolutions on 2D features in order to infer the accurate 3D pose in the video. Additionally, they also develop a semi-supervised method that deploys unlabeled video input data. They need the only camera intrinsic parameters instead of 2D annotations.

Ramakrishna in et al. [35] estimate 3D articulated pose where they design an over-complete dictionary comprised of vectors. They first categorize the training data into classes and then apply class-wise PCA to get their set of base vectors. They also enforce kinematic constraints utilizing information of the limb lengths. Fan et al. in [36] enhance the approach of [35] and introduces a model named Pose Locality Constrained Representation (PLCR) for estimation of 3D human poses. They build up a hierarchical human pose-tree through sub-clustering of human pose data. They develop a dictionary of the block-structural pose based on all the subspaces involved in the human pose-tree. Wang et al. [37] predict 3D human pose by exploiting the basis vectors combined with anthropometric constraints. For 2D poses estimation, they deploy a 2D pose estimator [38]. They optimize their objective function by utilizing the $L_{1}$ norm. Kanazawa et al. in [39] propose Human Mesh Recovery (HMR) that do not depend upon intermediate 2D image-based feature detections but predict the human shape and 3D articulated pose parameters from the pixels of the 2D input image directly.

Plenty of research works for 3D pose prediction [8,10,40,41,42,43,44] exploit prior knowledge available in MoCap dataset. Most of these data-driven methods need to reduce the curse of dimensionality to learn and train local models utilizing prior existing knowledge [20,40,42,45,46]. In [44], the authors propose a technique to animate the 2D characters in pictures using 3D MoCap dataset by fitting and deforming a 3D mesh model. The authors in [10] propose a dual-source approach—the annotated 2D poses and the accurate 3D MoCap data—predicting 3D pose by integrating both sources for efficient 3D human pose retrieval and reconstruction. The authors extend their work in [47] by improving the optimization process. Zhou et al. [48] propose an example-based approach that preserves locality similarity to infer 3D human pose. They extract the body parts having kinematic priors from the detected 2D pose and integrate them with 3D body parts to infer the 3D pose. In [49], authors estimate 3D pose by employing the process of memorization and warping the given 2D pose with a 3D pose library.

All approaches mentioned above or 3D estimator require the 2D pose estimation, which may be the joint locations, the silhouettes, or the limb edges. A few methods [50,51] label 2D joint location manually, a few approaches [37,46] exploit off-the-shelf 2D pose detector, and some works [52,53] deploy depth images for the prediction of 3D human poses. Another category of research work that estimates 3D articulated human pose utilizing Kinect cameras [54,55]. Several approaches have been seen in the literature that use the synthetic input data [7,8,15,21,35,36,43]. In [36], Fan et al., first project the pose with 18 joints into 2D space through a camera matrix generated by randomly selected camera parameters. They use CMU MoCap dataset. In [43], the authors create 2D synthetic videos using the HDM05 MoCap dataset, while [8] generate 2D synthetic poses for 3D reconstruction from the CMU MoCap dataset through a weak perspective camera model. In [7], the authors develop an image-dependable synthesis engine that generate a training dataset of *in-the-wild* synthetically. They compose the real images with synthetic 2D pose based on 3D MoCap data. They select 12,000 poses from the CMU MoCap dataset and sample 180 virtual views randomly. As a result, they create roughly 2M 3D/2D pose pairs. The authors in [56] annotate *in-the-wild* images by combining with ordinal depths of human joints, while [57] combine *in-the-wild* images with forward and backward information of every bone involved in the skeleton. Wang *et al.* [15] introduce a stereo-based artificial neural network to reconstruct the 3D poses from just two different viewpoints instead of deploying multi-view images. Their stereoscopic view synthetic subnetwork creates a 2D pose with right view from the given 2D pose with the left viewpoint. They generate the synthetic data through the unity toolbox in order to train the subnetwork. In our case, we develop synthetic 2D poses from the CMU MoCap dataset, use a 2D human pose detector [38] to predict 2D joint locations, and label 2D joint location manually from hand-drawn sketches.

## 3. Methodology

**Figure 1:**
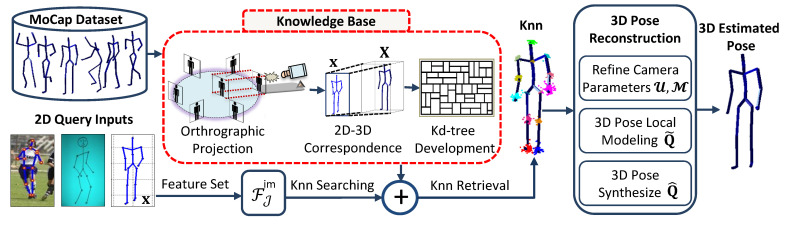
System architecture diagram. First a *knowledge-base* is developed for efficient 2D-3D correspondence, which involves the process of normalization, projection of the normalized 3D human poses onto the image-plane and the *k*d-tree development. The input to the system is either a synthetic 2D pose, an internet sport image or a hand-drawn sketch. A 2D feature set, i.e., $F_{J}^{im}\in \left\{F_{5}^{im},F_{7}^{im},F_{9}^{im},F_{11}^{im},F_{14}^{im}\right\}$ is used to search and retrieve Knn, which are further exploited in 3D reconstruction.

Our proposed methodology consists of multiple vital steps, which we discuss one by one in detail as below. The detailed version of the proposed system is presented in Figure 1.

### 3.1. Pose Skeleton Description

**Figure 2:**
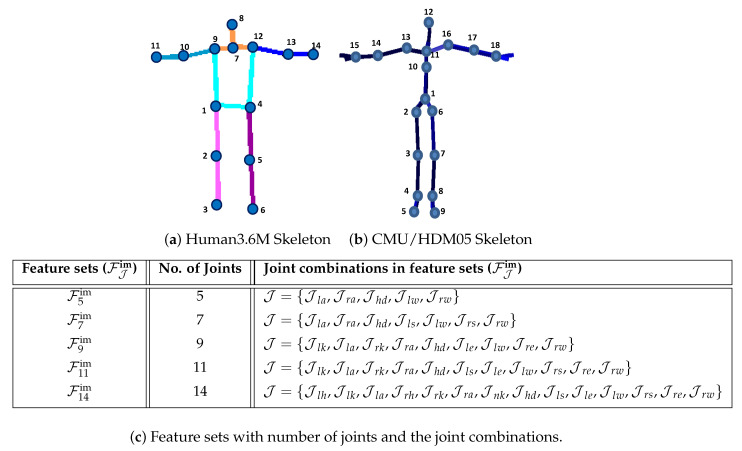
(**a**) The skeleton model S contains 14 joints for Human3.6M MoCap dataset and (**b**) 18 joints for CMU/HDM05 MoCap datasets, while (**c**) demonstrates all feature sets with different joint combinations.

We denote a 3D pose by X in Cartesian pose space R, which comprises of a set of N=18 number of joints for CMU [16] and HDM05 [17] MoCap datasets, while for Human3.6M [18], a 3D pose X consists of N=14 joints only. In case of 3D pose, the skeleton models S with all 14 or 18 number of joints are shown in Figure 2a and Figure 2b respectively. The skeleton model S with 18 joints (CMU [16] and HDM05 [17]) comprises of left and right hips ($J_{lh}$ and $J_{rh}$), left and right knees ($J_{lk}$ and $J_{rk}$), left and right ankles ($J_{la}$ and $J_{ra}$), left and right feet ($J_{lf}$ and $J_{rf}$), left and right shoulders ($J_{ls}$ and $J_{rs}$), left and right elbows ($J_{le}$ and $J_{re}$), left and right wrists ($J_{lw}$ and $J_{rw}$), head ($J_{hd}$), neck ($J_{nk}$), chest ($J_{ch}$), and the root joint ($J_{rt}$). The skeleton model S with 14 joints (Human3.6M [18]) consists of $J_{lh}$, $J_{rh}$, $J_{lk}$, $J_{rk}$, $J_{la}$, $J_{ra}$, $J_{ls}$, $J_{rs}$, $J_{le}$, $J_{re}$, $J_{lw}$, $J_{rw}$, $J_{hd}$, and $J_{nk}$.

Every joint $J\in R^{3}$ in the skeleton has *x*, *y*, and *z* components denoted as J(x), J(y), and J(z) respectively. A joint, e.g., the root joint, is expressed as $J_{rt}=\left[J_{rt}\left(x\right),J_{rt}\left(y\right),J_{rt}\left(z\right)\right]$. Finally, a 3D pose becomes $X={\left\{J_{i}\right\}}_{i=1}^{N}$, which shows the joint positions of the skeleton. In contrast, a pose with joint angle configurations in Quaternion pose space Q is represented by Q. A synthesized pose with joint positions in Cartesian pose space R is denoted by $\tilde{X}$, and with joint angle configurations is expressed as $\tilde{Q}$. A limb length between the parent joint $J_{p}$ and the child joint $J_{c}$ is represented by $L_{\left(p,c\right)}$ and the average limb length computed by taking an average of all limb lengths in the skeleton S is denoted as $L_{\left(p,c\right)}^{\prime }$. A 2D human pose is expressed by x. In case of 2D pose x, each joint $J\in R^{2}$ comprises of *x* and *y* components only, and the extracted image-based 2D feature sets are expressed by $F_{J}^{im}$ with different number of joints as described in Figure 2c, e.g., image-based 2D feature sets with 5 number of joints, $F_{5}^{im}$, consists of $J=\{J_{la},J_{ra},J_{hd},J_{lw},J_{rw}\}$.

### 3.2. Normalization

In pre-processing, we first normalize 3D poses in MoCap dataset in order to neutralize the differences that may exist in performing the same motion due to some additional information of translation and orientation. We are interested only in how the actions are executed and the posture are formed rather than focusing on where and at what view the postures are developed, i.e., the poses with translational and orientational information. The same two poses may have different coordinates due to this additional information of translation and orientation. In addition to all these, we also normalize the skeleton size of the performing actor.

#### 3.2.1. Translational Normalization

In translational normalization, we discard the translational information so that the 3D articulated human pose must hold on the center of the body’s mass, i.e., the root joint of the skeleton, at (0,0,0) coordinates in the Euclidean space. In case of Human3.6M [18] MoCap dataset, we compute the root joint of the skeleton by taking the average of the left and right hip joints,

(1) $$\left[\begin{array}{ccc} J_{rt}\left(x\right) & J_{rt}\left(y\right) & J_{rt}\left(z\right) \end{array}\right]=\left[\begin{array}{ccc} \frac{J_{lh}\left(x\right)+J_{rh}\left(x\right)}{2} & \frac{J_{lh}\left(y\right)+J_{rh}\left(y\right)}{2} & \frac{J_{lh}\left(z\right)+J_{rh}\left(z\right)}{2} \end{array}\right],$$

where $J_{lh}$ and $J_{rh}$ donate the left and right hip joints of the pose respectively. We subtract the coordinates of each joint from the coordinates of the root joint to eliminate the translational information,

(2) $$\hat{J_{i}}=J_{i}-J_{rt}\;\mathrm{and}\;i\in \left\{1,2,3,\ldots ,N\right\},$$

where $J_{rt}$ donates the root joint of the pose. After the translational normalization, all the poses transformed into position invariant coordinate system in Euclidean space.

#### 3.2.2. Orientational Normalization

In orientational normalization, we eliminate the orientation such that the pose has a frontal view only. All the joints of a pose are rotated by the *y*-axis, which is facing upward, such that the actor must face a frontal view with the positive *x*-axis while the hip joints must be parallel to the *z*-axis. For all that process, we first compute the rotation angle at which all the joints are rotated, utilizing the hip joints, $J_{lh}$ and $J_{rh}$, as,

(3) $$\theta =\arctan\left\{\frac{J_{lh}\left(x\right)-J_{rh}\left(x\right)}{J_{lh}\left(z\right)-J_{rh}\left(z\right)}\right\},$$

After having the estimated rotation angle, the *x*- and *z*-axes of each joint are turned by angle θ while the *y*-axis remains unchanged.

(4) $$\left[\begin{array}{cccc} J_{i}\left(x\right) & J_{i}\left(y\right) & J_{i}\left(z\right) & 1 \end{array}\right]={\left[\begin{array}{c} J_{i}\left(x\right)\\ J_{i}\left(y\right)\\ J_{i}\left(z\right)\\ 1 \end{array}\right]}^{T}\left[\begin{array}{cccc} \cos(\theta ) & 0 & \sin(\theta ) & 0\\ 0 & 1 & 0 & 0\\ -\sin(\theta ) & 0 & \cos(\theta ) & 0\\ 0 & 0 & 0 & 1 \end{array}\right]\;\mathrm{and}\;i\in \left\{1,2,3,\ldots ,N\right\},$$

This step is the same for both types of skeletons used in this work.

#### 3.2.3. Skeleton Size Normalization

We also normalize the skeleton’s size because the people vary in their heights. As a result, the coordinates of the same pose of two actors of different heights may differ from each other significantly. Each limb length of the skeleton is scaled up to an average limb length over a given entire population of the MoCap dataset. In the line of [58], taking the root joint, $J_{rt}$, as a parent joint $J_{p}$, all coordinates of its child joint $J_{c}$ are scaled in a way that the limb length that connects these joints is transformed to the average limb length $L_{\left(p,c\right)}^{\prime }$ as,

(5) $$J_{c}^{\prime }=\left(J_{c}+\alpha \cdot L_{\left(p,c\right)}\right),$$

(6) $$\alpha =\frac{L_{\left(p,c\right)}^{\prime }}{∥L_{\left(p,c\right)}∥}$$

We adjust each limb length recursively to average limb length based on the kinematic tree of the skeleton. Hence, the described procedure works for both skeleton types.

### 3.3. Search and Retrieval

In an exemplar-based reconstruction methodology, the critical component is an efficient and fast search and retrieval of Knn from the MoCap dataset. We have normalized 3D pose space, which includes only normalized 3D poses. We are dealing with a 2D image-based skeleton input query—that may be in the form of a synthetic 2D image generated through the projection of a 3D pose with some random camera parameters like in [35,36] or an image with 2D ground-truth pose, or an *in-the-wild* real picture or a 2D hand-drawn human pose, while our database consists of only 3D poses. Furthermore, our 2D input query pose has an absolute lack of knowledge like: (i) the camera parameters including depth information, (ii) the exact locations of the joints, (iii) the kinematic constraints of an image-based 2D skeleton, and (iv) the temporal coherence. We build an intermediate container named a *knowledge-base* to resolve this 2D-3D cross model search and retrieval problem. Through this, we not only resolve the issue of 2D-3D cross model retrieval but also make the process of search and retrieval more robust and convenient. We develop our *knowledge-base* by performing several steps like (i) we define 3D feature sets from the already developed normalized 3D poses and place them into the *knowledge-base* as the first component. (ii) We then create 2D pose space through an orthographic projection of 3D feature sets onto 2D image-based plane utilizing several virtual cameras. 24×7 virtual cameras are used, which have azimuth angles (0–345${}^{\circ }$) and the elevation angles (0–90${}^{\circ }$); both contain step size equal to 15${}^{\circ }$. (iii) We further re-scale the projected 2D normalized poses so as to fit it between some arbitrary scaling factor, i.e., [−1, 1]. We, then, also add these 2D normalized poses into our developed *knowledge-base*.

We design various feature sets based on skeleton joints’ subsets with different joint combinations to make similarity search fast and robust. These subsets of skeleton joints must hold the appropriate skeleton characteristics. According to [43,59], the most worthy and contributing joints in any type of pose are the end effectors (right/left hands and feet) and the head, which ensures not only the skeleton structure but also speed up the process of similarity search. As we are tackling 2D image-based input queries with no supporting cue, we may not rely on only the end effectors, but we must add up a few more joints. That’s why we devise several feature sets, i.e., $F_{5}^{im}$, $F_{7}^{im}$, $F_{9}^{im}$, $F_{11}^{im}$, $F_{14}^{im}$. The details about all these feature sets as well as the corresponding subsets of joints are presented in Figure 2c, while the performance of these feature sets is elaborated in Section 4.3.

With the *knowledge-base* with different normalized pose spaces, 2D image-based input query pose is given to the system. First, we normalize the 2D query pose by removing the translational information, i.e., we transform all the joints to their center of the mass by subtracting the root joints from all other joints in a 2D domain. We re-scale the normalized 2D pose in order to fit it according to the fixed arbitrary scaling factor. In short, we here normalize the 2D poses based on translation and the size of the skeleton of the pose. There is no need to perform the orientation normalization as we have already developed 2D normalized pose space by exploiting various virtual cameras to deal with. We define 2D feature sets from the normalized 2D input query pose. The 2D feature sets—either available in the *knowledge-base* or extracted from the 2D input query pose—both have become similar, equivalent, and comparable to each other for efficient 2D-3D cross domain search and retrieval of Knn. In the line of [41,43,59], we deploy *k*d-tree data structure for fast searching and retrieval of Knn.

### 3.4. Camera Parameters

We work with the weak perspective camera matrix M with *intrinsic* and *extrinsic* camera parameters, which is defined as,

(7) $$M=H\left[\begin{array}{c} R_{\left(\alpha ,\beta ,\gamma \right)}|T_{\left(x,y,z\right)} \end{array}\right],$$

where H denotes *intrinsic* and $\left[\begin{array}{c} R_{\left(\alpha ,\beta ,\gamma \right)}|T_{\left(x,y,z\right)} \end{array}\right]$ represent *extrinsic* camera parameters.

The *intrinsic* camera parameters H is expressed as,

(8) $$H=\left[\begin{array}{ccc} s_{x} & \kappa & \varepsilon_{x}\\ 0 & s_{y} & \varepsilon_{y}\\ 0 & 0 & 1 \end{array}\right]\left[\begin{array}{ccc} f & 0 & 0\\ 0 & f & 0\\ 0 & 0 & 1 \end{array}\right],$$

where $s_{x}$ and $s_{y}$ are the scales along *x* and *y*-axis, κ is the skew coefficient, $\varepsilon_{x}$ and $\varepsilon_{y}$ are the principal points along *x*-axis and *y*-axis, and *f* is the focal length. In our weak perspective camera model, we assume square-pixels, due to which the scaling factor $s_{x}$ becomes equal to $s_{y}$. The principal points $\varepsilon_{x}$ and $\varepsilon_{y}$ are considered image centers ideally, and κ is set to be zero. Ultimately, with these values, the above *intrinsic* camera parameters Equation (8) becomes,

(9) $$H=\left[\begin{array}{ccc} s & 0 & \varepsilon_{x}\\ 0 & s & \varepsilon_{y}\\ 0 & 0 & 1 \end{array}\right]\left[\begin{array}{ccc} f & 0 & 0\\ 0 & f & 0\\ 0 & 0 & 1 \end{array}\right]=\left[\begin{array}{ccc} \rho & 0 & \varepsilon_{x}\\ 0 & \rho & \varepsilon_{y}\\ 0 & 0 & 1 \end{array}\right],\;\rho =sf.$$

The *extrinsic* parameters $\left[\begin{array}{c} R_{\left(\alpha ,\beta ,\gamma \right)}|T_{\left(x,y,z\right)} \end{array}\right]$ involve 3 orientational variables (α, β and γ) and 3 translational variables ($T_{x}$, $T_{y}$ and $T_{z}$). Adopting the same formulation as in [6,21,35,36,37] where γ=0 and $T_{z}=1$. As a result, the projection matrix M becomes,

(10) $$M=\left[\begin{array}{ccc} \rho & 0 & \varepsilon_{x}\\ 0 & \rho & \varepsilon_{y}\\ 0 & 0 & 1 \end{array}\right]\left[\begin{array}{c} R_{\left(\alpha ,\beta ,0\right)}|T_{\left(x,y,1\right)} \end{array}\right]$$

Moreover, the translational parameters are taken as zero, considering that the centroid of the 3D pose coincides with the center of the mass of the 2D pose. The first two rows of M are orthogonal to each other since it is a weak perspective projection matrix.

In order to estimate the rest of the camera parameters, we formulate the two-fold nonlinear energy minimization method as,

(11) $$E_{cp}=\underset{U,M}{arg min}\left(aE_{a}+bE_{b}\right),$$

where U is a vector that contains the retrieved camera viewpoints. $E_{a}$ and $E_{b}$ donate the energy terms that we explain in the next paragraphs, while *a* and *b* are the related energy weights, which are the user-defined constants. We set the energy weights *a* and *b* equal to 0.45 and 0.55, respectively. These values are based on findings that we report in Section 4.2.4.

**Figure 3:**
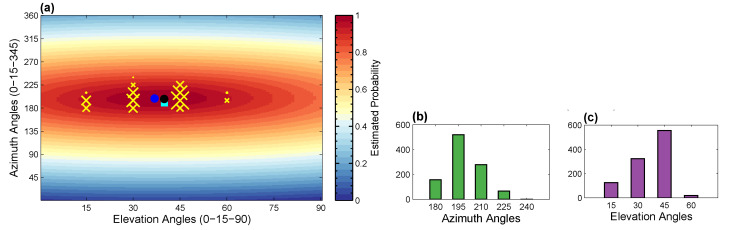
(**a**) An example to elaborate the estimation process of the camera viewpoints. The symbols *yellow cross* (×) represent the clusters of the viewpoints that we develop with the help of the retrieved nearest neighbors as well as from the projection of the normalized 3D poses at different view directions available in the *knowledge-base*. The symbol of the *bigger-sized yellow cross* (×) indicates a cluster of bigger size, i.e., the more number of similar poses are retrieved at this specific view angles compared to the symbol of the smaller cross (×). The *dark black circle* shows the results obtained using the symmetric square root kernel function. The *blue circle* expresses the simple arithmetic mean function, and the *cyan square* elaborates the ground-truth values. (**b**) shows the histograms of orientations developed on account of azimuth angles, while (**c**) shows the histograms of orientations for elevation angles. We have fixed the size of nearest neighbors equal to 1024 for this experiment.

In the first phase, we estimate the orientation information $R_{\left(\alpha ,\beta ,0\right)}$ from the retrieved nearest neighbors as well as from the projection of the normalized 3D poses at different view directions available in the *knowledge-base*. We consider this orientation estimation as the multi-label classification problem, where the number of classes is equal to 24×7 in correspondence to the virtual cameras (see Section 3.3). From the 2D input query pose, we retrieve the fixed size nearest neighbors with the information of view angles, i.e., the azimuth as well as the elevation angles. Each nearest neighbor executes the specific class of azimuth and elevation angle to which it belongs to. In this way, we develop the voting clusters and, ultimately, the histograms of orientations for azimuth and elevation angles separately based on majority voting. For example, in Figure 3a, the voting clusters for azimuth and elevation angles are expressed with *yellow cross* (×) symbols. Any specific virtual camera class with some higher votes results in a bigger cluster described with the *bigger-sized yellow cross* (×) symbol. We further illustrate the results of the voting clusters more precisely with histograms of orientations for azimuth angles as well as elevation angles, as reported in Figure 3b and Figure 3c respectively. To this end, we have the primary prediction for the camera viewpoints, considered the initialization for the estimation of the final camera parameters. Then, we optimize these voting clusters of the camera view directions utilizing the square-root kernel function as,

(12) $$E_{a}\left(U\right)=\sum_{k=1}^{K}\sqrt{∥U-V_{k}{∥}^{2}},$$

where the terms $V_{k}$ donates the k-th viewpoint observed during retrieval of fixed size nearest neighbors.

In the second phase, we further fine-tune the camera view directions and estimate the rest of the camera parameters. Having in hands the 2D joint locations of the input 2D query pose, the 3D K-nearest neighbors, and the primarily predicted camera viewpoints, we fine-tune the camera parameters as,

(13) $$E_{b}\left(M\right)=\sum_{k=1}^{K}\sqrt{\sum_{i\in J_{F}}{∥M\cdot X_{i,k}\;-x_{i}∥}^{2}},$$

where the notation $x_{i}$ represents the *i*th 2D joint location and $X_{i,k}$ is the *i*th 3D joint location of the *k*th nearest neighbor. For optimization, we employ the square root function as a symmetric kernel function because such a type of kernel-based representation is very appropriate to predict the arbitrarily shaped probability density with multiple peaks [41]. The multiple peaks may occur with the retrieval of multivariate Knn, as evident in Figure 3. We can still find the global minimum utilizing the square root kernel function even with numerous peaks compared to the simple arithmetic mean function. The two-fold nonlinear energy minimization method provides us a good initialization of camera view directions in the first phase and fine-tunes them further in the second phase. These initializations are the essential part in order not only to estimate the accurate camera parameters but also to speed up the minimization process. The orientation information U and the final camera matrix M are optimized using the Levenberg-Marquardt optimization algorithm using the nonlinear optimizer.

### 3.5. Pose Reconstruction

The proposed framework’s final goal is to infer a plausible 3D pose from the image-based 2D input query. For that purpose, we employ the prior existing knowledge already available in the MoCap dataset. We acquire this knowledge through our developed *knowledge-base* in the form of K nearest neighbors. We compute a linear local pose model using the retrieved similar poses $Q=\{Q_{1},\ldots ,Q_{K}\}$ by exploiting Principal Components Analysis (PCA),

(14) $$\tilde{Q}=CB+\mu ,$$

where the notation B denotes basis vectors, C is the current 3D human pose in PCA space, and μ is the mean pose of the Knn. We formulate the energy minimization problem as,

(15) $$\hat{Q}=\underset{\tilde{Q}}{arg min}\left(\omega_{p}E_{p}+\omega_{c}E_{c}\right).$$

where $E_{p}$ and $E_{c}$ represent the energy terms. $E_{p}$ measures and reduces the deviation from the retrieved Knn while $E_{c}$ decreases the projection error with 2D input query pose. $\omega_{p}$ and $\omega_{c}$ are the associated energy weights for energy terms $E_{p}$ and $E_{c}$ respectively. The optimizer for 3D estimation may be considered the bottleneck in the proposed approach’s performance efficiency. We here allocate each joint of the skeleton a specific weight that depends on the joint’s degree of freedom (*dof*). We suppose that the joints containing higher *dof* have a deep impact on the body parts’ movements and contribute more to the joints having lower *dof*. We will validate this assumption in Section 4.2.3. On that basis, we assign higher weights to those specific joints having higher *dof*. For example, the ball-and-socket joints having 3 *dof* are allocated higher weights than the hinge joints with just only 1 *dof*. We further normalize the assigned weights with min-max normalization and express them with a vector as $w=\{w_{1},\ldots ,w_{J}\}$. We employ the gradient descent based energy minimization method.

#### 3.5.1. Retrieved Pose Error

As we are dealing with a large heterogeneous MoCap dataset and the input query comprises a subset of the 2D pose’s joints only, we work with combination of joint angle configurations and the joint positions to produce 3D plausible results. On this basis, we penalize the deviation of the synthesized human pose from the retrieved K nearest neighbors not only in the Quaternion 3D pose space Q but also in the Cartesian 3D pose space R. In this context, we design the energy term as,

(16) $$E_{p}=\omega_{pa}E_{pa}+\omega_{pp}E_{pp},$$

where the notations $E_{pa}$ and $E_{pp}$ are the energy terms corresponding to Quaternion pose space Q and the Cartesian pose space R respectively. The symbols $\omega_{pa}$ and $\omega_{pp}$ are the associated weights with $E_{pa}$ and $E_{pp}$ respectively. For $E_{p}$, we consider all the joints of the skeleton model S.

The first part of the energy term, $E_{pa}$, deals with joint angle parameterizations in the quaternion pose space Q and compels the synthesized 3D pose $\tilde{Q}$ to be according to the prior existing knowledge in the MoCap dataset,

(17) $$E_{pa}\left(\tilde{Q}\right)=\sum_{k=1}^{K}\sqrt{\sum_{i\in J}{∥w_{i}\cdot \left(Q_{i,k}-{\tilde{Q}}_{i}\right)∥}^{2}},$$

where $w_{i}$ is the weight for each joint.

The second part of the energy term, $E_{pp}$, directly imposes the 3D joint locations of the synthesized 3D pose in the cartesian pose space R to be according to the retrieved K nearest neighbors,

(18) $$E_{pp}\left(\tilde{X}\right)=\sum_{k=1}^{K}\sqrt{\sum_{i\in J}{∥w_{i}\cdot \left(X_{i,k}-f\left({\tilde{Q}}_{i},S_{i}\right)\right)∥}^{2}},$$

where f represents the forward-kinematics function which converts joint angle configurations of the synthesized 3D pose $\tilde{Q}$ into joint positions, $\tilde{X}$. The symbol S denotes the skeleton model, developed through recursively transforming each limb length $L_{\left(p,c\right)}$ to average limb length $L_{\left(p,c\right)}^{\prime }$ based on the skeleton’s kinematic tree. The notation $X_{i,k}$ is the *i*th joint location of the retrieved kth similar poses.

#### 3.5.2. Projection Control Error

This energy term minimizes the projection error and fits the synthesized pose to 2D image query pose by utilizing the estimated camera parameters in the projection matrix M,

(19) $$E_{c}\left(\tilde{X}\right)=\sqrt{\sum_{i\in J_{F}}∥w_{i}\cdot \left(M\cdot f\left({\tilde{Q}}_{i},S_{i}\right)-x_{i}\right){)∥}^{2}}.$$

We consider here only those joints, $J_{F}$, which participate in creating the specific feature set, i.e., $F_{11}^{im}$, and are used in search and retrieval of Knn.

## 4. Experiments

We evaluate our proposed approach thoroughly on different types of MoCap datasets qualitatively as well as quantitatively, namely CMU [16], HDM05 [17], and Human3.6M [18] MoCap datasets. Moreover, we test our proposed system on different categories of input testing datasets: synthetic 2D images, annotated 2D images, the *in-the-wild* internet real images, or even the hand-drawn sketches. Similar to [36], we deploy the skeleton that consists of 18 joints for CMU [16] and HDM05 [17] MoCap datasets. These joints are head, neck, shoulders, chest, elbows, wrists, root, hips, knees, ankles, feet, as reported in Figure 2b. The body skeleton, in case of Human3.6M [18] MoCap dataset, comprises of 14 joints, i.e., head, neck, hips, knees, ankles, shoulders, elbows, and wrists as described in Figure 2a.

For the error measurement, we follow the same protocol as in [8,36], i.e., the normalized *reconstruction error* as well as the *reconstruction rate*. In case of the normalized *reconstruction error*, the error is computed at every joint by measuring the Euclidian distance between 3D locations of each joint of the reconstructed human pose and the ground-truth human pose. We then select the joint error that shows the highest reconstruction error compared to all 18 joints. It is further normalized by taking the fraction multiplication with the backbone length (the distance between the chest joint and the root joint). For multiple 2D input query images, we compute an average of the *reconstruction error* for all input query images and name it as *average reconstruction error*, shortly *recon-err*. In case of *reconstruction rate*, it is defined as the percentage of the test input query images with lowest *reconstruction error* subject to some specified threshold, i.e., 0.3 in the line of [8,36]. We Procrustes fit the 3D reconstructed human pose with the ground-truth 3D human pose before computing the final reconstruction error as in [8,35,36]. We deploy these error measurements when evaluating our approach on CMU [16] and HDM05 [17] MoCap datasets. For evaluation on Human3.6M [18] MoCap dataset, we employ the *3D pose error* as defined in [10,46,47], where the predicted pose is aligned first to the ground-truth pose by the rigid transformation, and then the mean 3D Euclidean joint error is computed.

### 4.1. Datasets

We first discuss the datasets we deploy in this paper to conduct plenty of experiments to evaluate the developed system. We elaborate on the MoCap datasets that we use to infer the missing 3D information and the different kinds of testing input datasets that we use for evaluation purposes.

#### 4.1.1. Mocap Datasets

We employ three popular and challenging MoCap datasets, named CMU [16], HDM05 [17], and Human3.6M [18] MoCap dataset, all are available publicly. In case of CMU dataset, the Vicon system with 12 infrared MX-40 cameras is used in order to capture the human motions at a 120 Hz sampling rate [16]. It is recorded by 144 actors performing different types of motions, including gymnastics and other interesting physical activities. We work with roughly ${}^{1}\;{/}_{3}$ of the CMU MoCap dataset because of the limited memory capacity.

The second dataset used in this research paper is HDM05 [17] that has about 2337 number of motions with 130 categories performed by five different performing actors. The motions are recorded using a Vicon MX system with 120 Hz sampling rate, consisting of 12 high-resolution cameras.

The third MoCap dataset, Human3.6M [18], is also a large-scale indoor MoCap dataset that provides 3D annotations. It contains 3.6M 3D human poses with their corresponding images, performed by 11 professional actors. In this dataset, we deal with 15 classes, i.e., direction (dir.), discussion (disc.), eating (eat), greeting (greet), talking on phone (ph.), pose, making purchase (pur.), sit, sit down (sitD.), smoking (smoke), taking photo (photo), waiting (wait), walking (walk), walking dog (walkD.) and walking together (walkT.).

We first down-sample both CMU and HDM05 MoCap datasets from sampling rate 120 Hz to 30 Hz. Consequently, we obtain roughly 360K number of poses for CMU MoCap dataset and 380K number of poses for HDM05 MoCap dataset. We further categories these datasets into four different experimental protocols and scenarios such as ${\mathrm{MDS}}_{cmu}$, ${\bar{\mathrm{MDS}}}_{cmu}$, ${\mathrm{MDS}}_{hdm}$ and ${\mathrm{MDS}}_{h3.6m}$.

#### ${\mathrm{MDS}}_{cmu}$

It consists of all 3D human poses of CMU Mocap Dataset, excluding only those human poses used to create a 2D synthetic input testing dataset. Moreover, we also discard absolutely all motions from which we make even a single 2D synthetic input query image. In this way, the dataset ${\mathrm{MDS}}_{cmu}$ becomes entirely free of overlaps with any 2D input query image.

#### ${\bar{\mathrm{MDS}}}_{cmu}$

It also comprises all 3D human poses of the CMU Mocap dataset; however, we eliminate all those motions completely, from which we create even a single 2D synthetic input query image. Additionally, we also discard all motion sequences in which the same performing actor appears as in the 2D synthetic input query image. Therefore, this dataset is free of the motion sequences and the performing actors relevant to the input query pose.

#### ${\mathrm{MDS}}_{hdm}$

This dataset is developed using HDM05 motion capture sequences. It contains all the poses, i.e., 380K number of poses, as we generate no 2D synthetic input query image using this MoCap dataset. In other words, this MoCap dataset is absolutely free from any input query pose.

#### ${\mathrm{MDS}}_{h3.6m}$

On the Human3.6M MoCap dataset [18], we follow up the same protocol as in [10,47,48,49] and use six different subjects, i.e., S1, S5, S6, S7, S8, and S9, for developing the training dataset. In the line of [10,47], we discard every other pose if the average Euclidean distance between two consecutive poses is less than 1.5 mm. As a result, the Human3.6M MoCap dataset is reduced to 380K 3D human poses. For testing, we employ every 64th frame of the subject S11.

#### 4.1.2. Input Datasets

We evaluate our proposed system thoroughly on three different types of input query dataset.

#### Synthetic 2D Dataset

For quantitative analysis of the proposed framework, we follow the same protocol as mentioned in [8,35,36] and create synthetic 2D input testing datasets from the CMU MoCap dataset using a camera matrix with random parameters. We also select only those action classes, as in [8,36], i.e., walking, running, boxing, jumping, and climbing. We refer to synthetic 2D input testing datasets as ${\mathrm{SDS}}_{1}$, which contains 43,809 numbers of 2D synthetic input query poses with 25 subjects as reported in Table 1. It is the same dataset as mentioned in [8], and it is large enough compared to the dataset [36], which consists of 29,336 synthetic poses with 23 subjects.

We also generate a mini input testing dataset ${\mathrm{SDS}}_{2}$ that is the subset of the input testing dataset ${\mathrm{SDS}}_{1}$. We randomly select 3500 synthetic 2D input images from almost all action categories. We develop this test dataset ${\mathrm{SDS}}_{2}$ just for tuning the parameters.

#### PARSE Dataset

For qualitative evaluation, we deploy here in this paper PARSE dataset [19,60], which consists of *in-the-wild* internet real sports images.

#### Hand-Drawn Sketches Dataset

We also assess our proposed framework qualitatively on 2D hand-drawn human poses. We created 2D sketches for human poses by hand. It is very challenging to infer plausible 3D poses from 2D hand-drawn sketches since most of the drawings do not meet the kinematic and anthropometric constraints inherent in human beings’ natural poses. The results section will show that our proposed system performs very well in such type of most challenging input queries.

### 4.2. Parameters

We perform several preliminary experiments to tune and adjust the values for the parameters. We elaborate on these experiments for parameter tuning below.

#### 4.2.1. Principal Components

**Figure 4:**
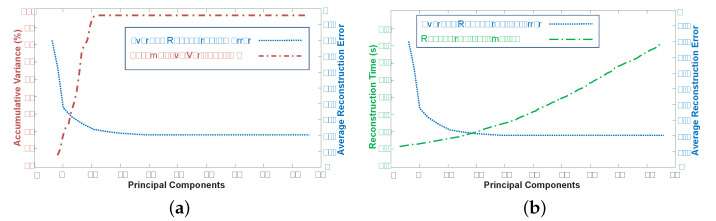
(**a**) Accumulative variance (%) as well as average reconstruction error for the dynamic selection of the number of principal components. (**b**) Reconstruction time and the average reconstruction error for different number of principal components.

We apply PCA in order to compute the linear local model in a linear subspace with lower dimensionality. The energy minimization is much faster in a restricted lower-dimensional space. Thus, it is worth the overhead of computing the PCA for each pose. To handle the trade off between potentially lower accuracy introduced by the PCA and faster optimization times, we dynamically decide for the number of principal components (eigenposes). We calculate the captured variance of the retrieved similar poses for every given 2D input query pose separately. As a result, the number of eigenposes varies for every 2D input query pose. We select the lowest number of eigenposes, such that the accumulative variance is larger than 99%. Based on this criterion, we observe that the number of eigenposes selected is within the range of 14 to 20. We show an example in Figure 4a, where the accumulative variance and the average reconstruction error are computed for changing numbers of principal components. We can see that for this specific case, the average reconstruction error converges for more than 18 eigenposes while the accumulative variance converges, too. Figure 4b shows the reconstruction error and the computation time for the varying number of principal components. Hence, the advantage of the local dimensionality reduction in terms of computations times becomes more clear.

#### 4.2.2. Nearest Neighbors

**Figure 5:**
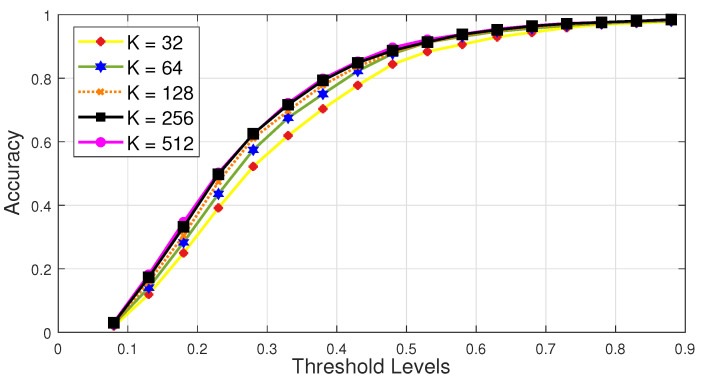
Comparing different values of K, i.e., the numbers of nearest neighbors needed to reconstruct 3D human pose. We conduct this experiment on the input testing dataset ${\mathrm{SDS}}_{2}$ and use average reconstruction error (*recon-err*) with varying threshold values.

We conduct experiments to fix some appropriate value for K, i.e., the total number of nearest neighbors. We perform this experiment by fixing the values for K as, $2^{5}=32$, $2^{6}=64$, $2^{7}=128$, $2^{8}=256$, and $2^{9}=512$. Then, we evaluate the system’s performance at different threshold levels, like, {0.1,0.2,0.3,…,0.9}. From the results as reported in Figure 5, we have found that the proposed system executes its best results comparatively at almost all threshold levels when the value for K =256. We have performed this experiment on the input testing dataset ${\mathrm{SDS}}_{2}$. We fix the value of K=256 for all other experiments to evaluate our proposed system.

#### 4.2.3. Joint Weights

**Figure 6:**
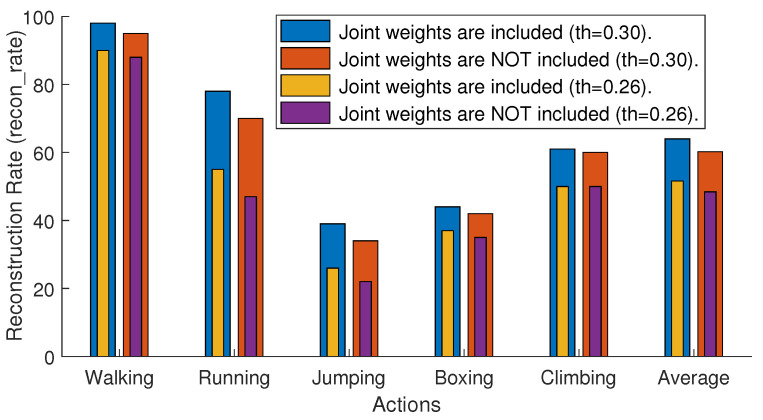
The overall influence of the joint weights w. We carry out this experiment on input testing dataset ${\mathrm{SDS}}_{2}$, and the error measurement, *recon-rate* with thresholds 0.3 and 0.26, is used.

As mentioned earlier in Section 3.5, we assign weight to each skeleton’s joint according to the degree-of-freedoms. We experiment to see the overall influence of the joint weights on our proposed architecture. We compute *recon-rate* with and without joint weights at two different threshold levels, i.e., 0.3 and 0.26. The results mentioned in Figure 6 show that the usage of the joint weights increases accuracy significantly.

#### 4.2.4. Energy Weights

**Figure 7:**
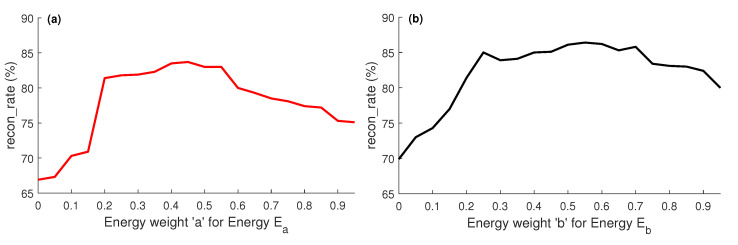
Impact of the weighted energy terms, $E_{a}$ and $E_{b}$ is reported in (**a**), and (**b**) respectively.

We first evaluate the impact of the energies $E_{a}$ and $E_{b}$ (Section 3.4) by allocating different weights to them, ranging from 0.0 to 0.9. The results reported in Figure 7 show that for the energy weights a=0.45 and b=0.55, we obtain the best reconstruction results (*recon-rate*), which is an evident that the energies $E_{a}$ and $E_{b}$ with those weights play a vital role in the estimation of the accurate camera parameters.

**Figure 8:**
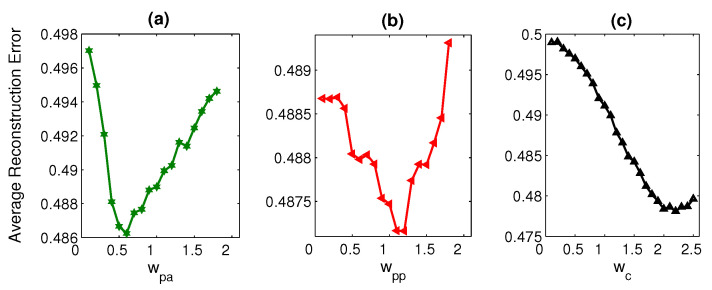
Impact of the weighted energy terms, $E_{pa}$, $E_{pp}$, and $E_{c}$, is reported in (**a**), (**b**), and (**c**), respectively.

We also perform experiments to examine the influence of the energy terms $E_{pa}$, $E_{pp}$, and $E_{c}$, which we employ in the reconstruction process (Section 3.5). We assign different weights to these energy terms starting from 0 to some specific value. While investigating the weights for an energy term, we keep the weights for other energy terms fixed to some particular values. We have concluded from the results presented in Figure 8 that at the energy weights such as $\omega_{pa}=0.8$, $\omega_{pp}=1.4$, and $\omega_{c}=1.8$, these energy terms contribute substantially, and the overall performance of the proposed methodology increases significantly.

It is also evident from Table 3 that the energy minimization function with multiple energy terms and the dynamic number of principal components improve accuracy convincingly as compared to the PCA-based method with fixed principal components, PC = 18.

#### 4.2.5. Virtual Cameras

We have generated several virtual cameras through which the MoCap dataset is projected onto the image plane to deal with 2D-3D cross-domain similarity and retrieval issues (see Section 3.3). We experiment to see the overall impact of the virtual cameras on the proposed framework’s efficiency in two steps,

- First, we create virtual cameras just by fixing the elevation (elv) angles (0–15–90${}^{\circ }$) and varying the azimuth (azm) angles (0–345${}^{\circ }$ with 15${}^{\circ }$, 25${}^{\circ }$, 35${}^{\circ }$, 45${}^{\circ }$, and 60${}^{\circ }$ step sizes). As a result, we create several virtual cameras as, {(24×7),(15×7),(11×7),(8×7),(6×7)} with azimuth and elevation incremental step sizes $\{(\mathrm{azm}\left(15^{\circ }\right)$, $\mathrm{elv}\left(15^{\circ }\right)),\left(\mathrm{azm}\left(25^{\circ }\right),\mathrm{elv}\left(15^{\circ }\right)\right),\left(\mathrm{azm}\left(35^{\circ }\right),\mathrm{elv}\left(15^{\circ }\right)\right),\left(\mathrm{azm}\left(45^{\circ }\right),\mathrm{elv}\left(15^{\circ }\right)\right),$$(\mathrm{azm}\left(60^{\circ }\right),$$\mathrm{elv}(15^{\circ }))\}$ respectively.
- Second, we generate virtual cameras by fixing the azimuth (azm) angles (0–15–345${}^{\circ }$) and by varying the elevation (elv) angles (0–90${}^{\circ }$ with 15${}^{\circ }$, 30${}^{\circ }$, and 45${}^{\circ }$ step sizes). Consequently, several virtual cameras are created as, {(24×7),(24×4),(24×3),(8×7)} with azimuth and elevation incremental step sizes $\{\left(\mathrm{azm}\left(15^{\circ }\right),\mathrm{elv}\left(15^{\circ }\right)\right),$$(\mathrm{azm}\left(15^{\circ }\right),\mathrm{elv}\left(30^{\circ }\right)),$$\left(\mathrm{azm}\left(15^{\circ }\right),\mathrm{elv}\left(45^{\circ }\right))\right\}$ respectively.

**Figure 9:**
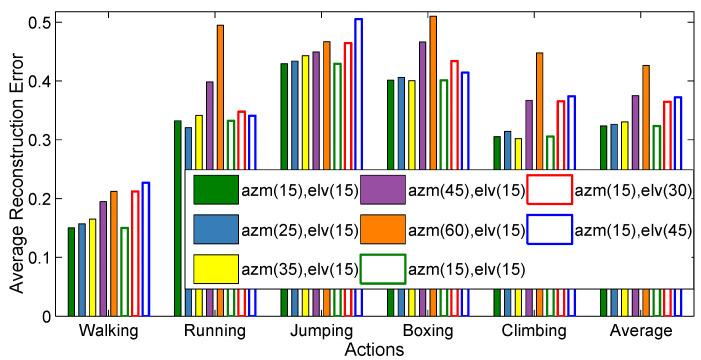
The influence of the number of virtual cameras. The filled colored bars represent the average reconstruction error based on the sampling of the azimuth angles, i.e., $\{\left(\mathrm{azm}\left(15^{\circ }\right),\mathrm{elv}\left(15^{\circ }\right)\right),\left(\mathrm{azm}\left(25^{\circ }\right),\mathrm{elv}\left(15^{\circ }\right)\right),\left(\mathrm{azm}\left(35^{\circ }\right),\mathrm{elv}\left(15^{\circ }\right)\right),\left(\mathrm{azm}\left(45^{\circ }\right),\mathrm{elv}\left(15^{\circ }\right)\right),(\mathrm{azm}\left(60^{\circ }\right),$$\mathrm{elv}(15^{\circ }))\}$, which are equivalent to {(24×7),(15×7),(11×7),(8×7),(6×7)} number of virtual cameras respectively. On the other hand, the unfilled colored bars show the average reconstruction error based on the sampling of the elevation angles, i.e., $\left\{\left(\mathrm{azm}\left(15^{\circ }\right),\mathrm{elv}\left(15^{\circ }\right)\right),\left(\mathrm{azm}\left(15^{\circ }\right),\mathrm{elv}\left(30^{\circ }\right)\right),\left(\mathrm{azm}\left(15^{\circ }\right),\mathrm{elv}\left(45^{\circ }\right)\right)\right\}$, which generate {(24×7),(24×4),(24×3),(8×7)} number of virtual cameras respectively.

The results in Figure 9 elaborate that when the step sizes for the azimuth angles (azm) increase, the error (*recon-err*) increases accordingly. The same response is noticed in case of the elevation angles. More precisely, more virtual cameras are deployed, the average reconstruction error decreases correspondingly. We use virtual cameras equal to (24×7) for the rest of the experiments.

### 4.3. Search and Retrieval

For searching and retrieval of Knn from the developed *knowledge-base*, we define a variety of feature sets through different combinations of skeleton joints as described in Figure 2b. We perform various types of experiments to analyze all these feature sets based on nearest neighbors retrieval, systems’s accuracy, memory consumption, and the time complexity. For all these experiments, we deploy input testing dataset ${\mathrm{SDS}}_{2}$. We elaborate on these experiments and discuss the results one by one as under.

**Figure 10:**
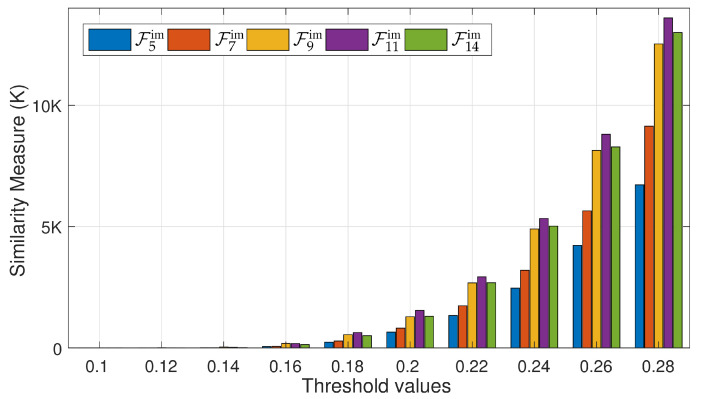
A comparison between different feature sets, i.e., $F_{5}^{im}$, $F_{7}^{im}$, $F_{9}^{im}$, $F_{11}^{im}$, and $F_{14}^{im}$, in terms of robust nearest neighbors retrieval—the similarity measure. We fix the Knn equal to 256 and experiment on 1500 synthetic 2D poses, selected randomly. Consequently, it becomes 256×1500 number of target poses. For this experiment, we use average reconstruction error, *recon-err*, with various thresholds.

In the first experiment, we evaluate all these feature sets in terms of similarity search and retrieval, i.e., the retrieval of Knearest neighbors when the value of K is fixed to be 256. For this experiment, we randomly select 1500 input 2D poses from the input testing dataset ${\mathrm{SDS}}_{2}$ and are provided to the system as query. For 2D input query poses, we search for and retrieve K nearest neighbors using all the feature sets $F_{5}^{im}$, $F_{7}^{im}$, $F_{9}^{im}$, $F_{11}^{im}$, and $F_{14}^{im}$ separately one by one. As a result, we retrieve poses equal to 256×1500 for every feature set. We then compare these feature sets in terms of the retrieved nearest neighbors. More precisely, we count only those nearest neighbors that yield low *recon-err* at some specific threshold. Figure 10 demonstrate that the feature set, $F_{11}^{im}$, retrieves a very good number of nearest neighbors as compared to all other feature sets at almost all threshold values.

**Figure 11:**
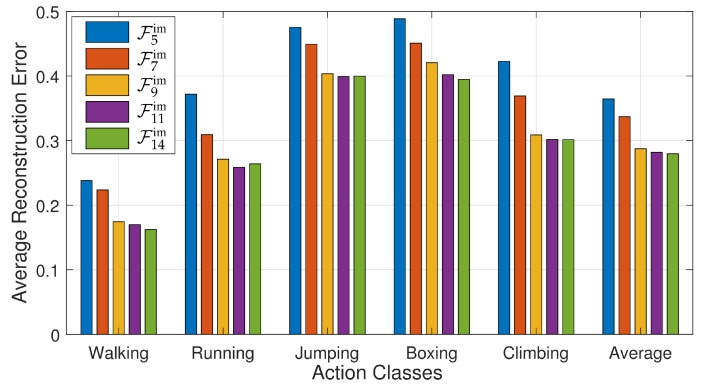
A comparison between feature sets, i.e., $F_{5}^{im}$, $F_{7}^{im}$, $F_{9}^{im}$, $F_{11}^{im}$, and $F_{14}^{im}$, based on *recon-err*, for all five action classes and the average results obtained by computing the average of all actions.

In the second experiment, we analyze all feature sets’ performance based on the reconstruction of 3D articulated poses for different types of action poses. Moreover, we also consider the average results of all these action categories. We conclude from the results shown in Figure 11 that both feature sets, $F_{11}^{im}$ and $F_{14}^{im}$, show their performance very well comparatively not only for all action categories but also on an average taken from the results of all the action classes.

In the third experiment, we measure the developed feature sets’ performance in terms of time consumption as well as memory allocation. The results are shown in Table 2, which illustrates that the feature set $F_{5}^{im}$ takes less time than all other feature sets in the process of retrieval and reconstruction. However, the difference in time is not relevant as the energy minimization is the time-consuming factor. Furthermore, the time spent on constructing the *knowledge-base* and the *k*d-tree is less critical, since both are the pre-processing steps and are performed just once to retrieve the nearest neighbors. As expected, the feature sets containing more joints require more memory allocation comparatively in terms of memory allocation. For example, the feature set $F_{14}^{im}$ requires more memory in comparison to other feature sets.

In the end, we conclude from these experiments that the feature set $F_{11}^{im}$ is the best choice on account of similarity retrieval, reconstruction accuracy, time consumption, and memory allocation. It is in contrast to [43,59], where the authors claim that the feature sets $F_{5}^{im}$ (head and four end effectors) is the best choice for the similarity retrieval. No doubt that $F_{5}^{im}$ has less time complexity and needs less memory allocation, but the system’s accuracy drops substantially, as apparent in results shown in Figure 10 and Figure 11. The choice of an appropriate feature set is a trade-off between the system’s accuracy and time-memory complexities. In the end, we select and recommend the feature set, $F_{11}^{im}$, that yields not only more system’s accuracy but also very appropriate with respect to time and memory complexities. We implement these experiments using a single-threaded MATLAB, 64-bit operating system (Window 10 pro), 32 GB RAM, and a Core 12 @ 3.20 GHz processor.

### 4.4. Quantitative Evaluation

For quantitative analysis of our proposed approach, we employ the testing input dataset ${\mathrm{SDS}}_{1}$, which comprises 43,809 synthetic 2D poses as reported in Table 1. For that purpose, we design and conduct a pool of experiments, which we illustrate as follows.

#### 4.4.1. Evaluation on ${\mathrm{MDS}}_{cmu}$

In the first experiment, we deploy dataset ${\mathrm{MDS}}_{cmu}$ as knowledge prior. The results reported in Table 3a illustrate that our approach convincingly outperforms the PCA-based method (with fixed principal components, PC = 18) and other state-of-the-art approaches [8,35,36], for almost all five action categories on both evaluation metrics, i.e., *recon-err* as well as *recon-rate*.

#### 4.4.2. Evaluation on ${\bar{\mathrm{MDS}}}_{cmu}$

In the second experiment, we use dataset ${\bar{\mathrm{MDS}}}_{cmu}$ as a knowledge prior. Our proposed approach performs best for all types of action classes except for the climbing action, as shown in Table 3b. In case of climbing motion sequences, the error increases because ${\bar{\mathrm{MDS}}}_{cmu}$ includes only very few examples of climbing motions.

#### 4.4.3. Evaluation on ${\mathrm{MDS}}_{hdm}$

In the third experiment, the HDM05 MoCap dataset is used to get prior knowledge from the MoCap dataset. In this experimental setup, the query input is from the CMU dataset, ${\mathrm{SDS}}_{1}$, and the MoCap dataset is HDM05, ${\mathrm{MDS}}_{hdm}$. The results reported in Table 3c show that the error increases because of the skeleton discrepancies between HDM05 and CMU MoCap datasets. Moreover, the error for boxing action is relatively high since the HDM05 dataset does not have boxing motion sequences but contains very few punching motion examples. Even having such types of challenges, our proposed system still executes very competitive results.

In terms of time, our proposed approach is more efficient as it takes only 0.668 s. per 2D input image for the process of retrieval and reconstruction with the feature set $F_{11}^{im}$ as mentioned in Table 2. In comparison, the other state-of-the-art method [35] needs 5 s/image to infer.

#### 4.4.4. Evaluation on ${\mathrm{MDS}}_{h3.6m}$

For evaluation on the Human3.6M MoCap dataset [18], we follow the same protocol as in [10,47,48,49] and deploy 3D pose error [10,46,47] for the error measurement. The results reported in Table 4 show that our proposed pipeline executes outstanding results compared to other state-of-the-art approaches. Our proposed method outperforms almost all other state-of-the-art techniques on most of the classes as well as on average results. A very few classes like in sit, sitDown, and walkDog, where other state-of-the-art techniques show less reconstruction error than our method, our approach still produces very competitive results, as shown in Table 4.

#### 4.4.5. Evaluation on Noisy Input Data

**Figure 12:**
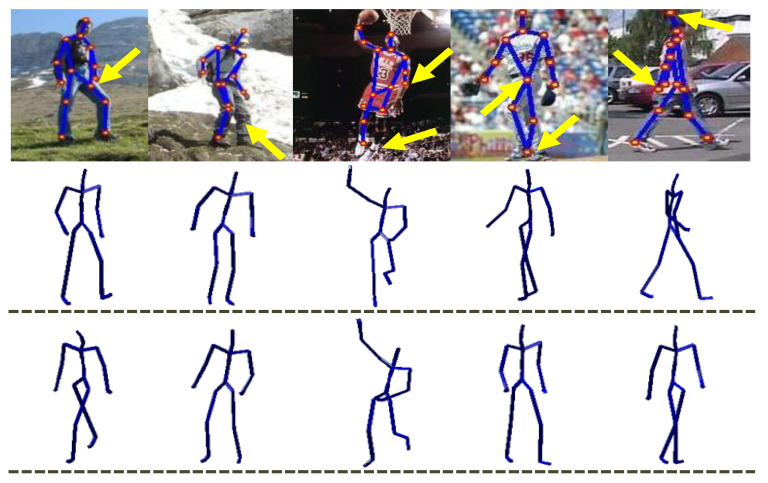
Qualitative evaluation of the proposed architecture on noisy input data—A few 2D input query images with joint outliers **(first row)** are given to the system while the final 3D reconstructed poses are shown in **second row** and **third row** at two different view angles.

In real life, the estimated 2D pose and the joint locations from images are often inaccurate and noisy. We also analyze the proposed approach with noisy input data as well. In the line of [36], we generate noisy query input with Gaussian white noise with different levels of standard deviation σ like 0.0 (no noise), 0.1, 0.2, 0.3, and 0.4. Furthermore, we normalize the noise before adding it up into synthetic input query 2D poses. Our proposed methodology shows more resistance to noise comparatively, as reported in Table 5. It is further evident from Figure 12, where our proposed system executes very acceptable qualitative results even when the input query 2D pose consists of erroneous 2D joint locations. Moreover, we evaluate our method on hand-drawn sketches, which contains a wildly inaccurate skeleton that does not hold even anthropometric regularity. On hand-drawn sketches even, our system produces very plausible results, as shown in Figure 14.

### 4.5. Qualitative Evaluation

For qualitative evaluation of our proposed methodology, we conduct several experiments on the *in-the-wild* real images and on 2D hand-drawn human poses, which we create manually. We discuss the experiments and the results one by one below.

#### 4.5.1. Real Images of Parse Dataset

**Figure 13:**
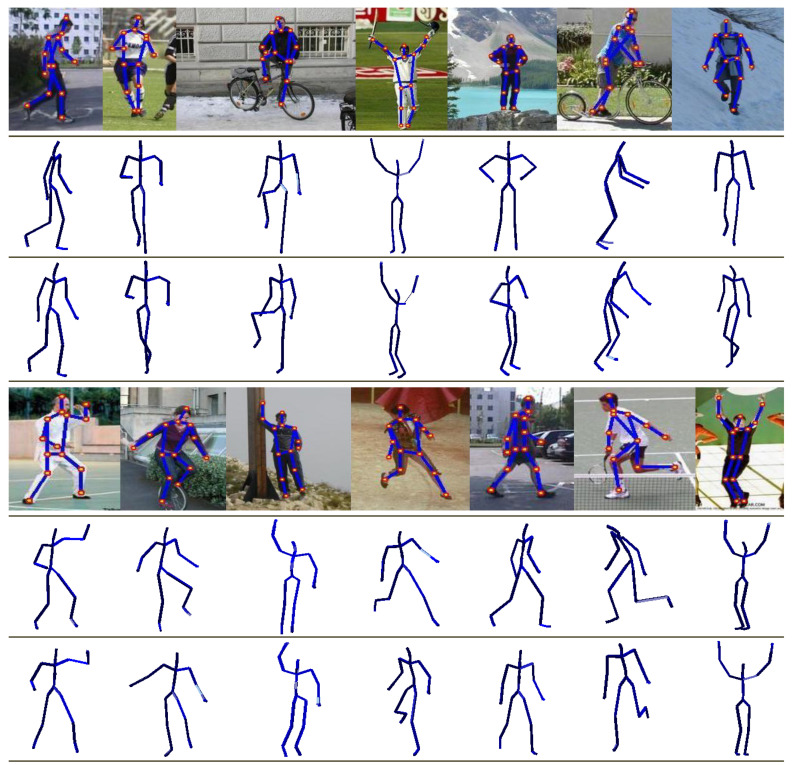
Qualitative results on the *in-the-wild* internet real images taken from PARSE dataset [19]. The **first rows** show the input *in-the-wild* real images with 2D joint annotations estimated through [38]. The **second** and the **third rows** represent the relevant 3D reconstructed human poses at two different view angles.

We deploy the PARSE dataset [19] for qualitative performance evaluation of our proposed system on the *in-the-wild* real images, as described earlier in Section 4.1. In order to estimate 2D joint locations from the given *in-the-wild* real image, we utilize an off-the-shelf 2D part-detector method [38] that detects an overall 2D skeleton from the given image, while other approaches [35,36] label the 2D joint positions manually. In our case, the 2D part-detector approach [38] yields a more noisy 2D skeleton from *in-the-wild* real images comparatively. A few qualitative reconstruction results of our proposed system are shown in Figure 13. These results demonstrate that even with noisy 2D estimated joint positions, our methodology produces very plausible 3D reconstruction poses.

#### 4.5.2. Hand-Drawn Sketches

**Figure 14:**
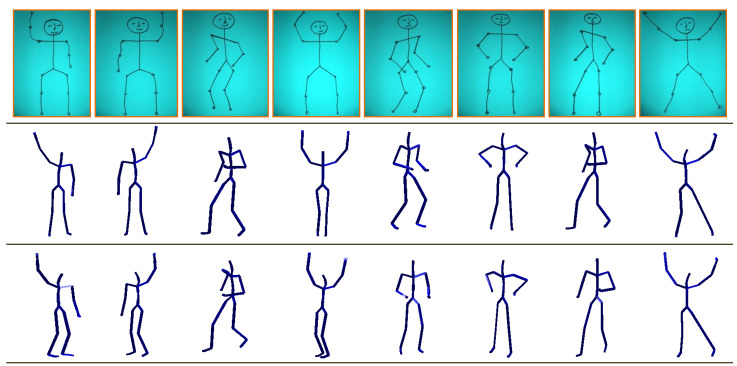
Qualitative results on the hand-drawn sketches created manually. The **first row** shows the input hand-drawn sketches. The **second** and the **third rows** are the relevant 3D reconstructed human poses at two different view angles.

We also make a qualitative evaluation of our approach on 2D hand-drawn sketches of human poses, which we draw manually for different action classes. The robust and plausible reconstruction of 3D articulated human poses from just only the 2D hand-drawn poses may be considered as the most challenging problem based on the following reasons: (i) the lack of the anthropometric and kinematic constraint in the skeleton of the 2D hand-drawn human poses; (ii) the variations in the limbs’ lengths; (iii) the unnatural movements of the body parts etc. We first annotate the 2D joint locations manually of the given 2D hand-drawn pose and then input it to the developed system as a query. The results presented in Figure 14 explore that our proposed method yields very acceptable 3D reconstructed human poses even on such an ambiguous and noisy input.

### 4.6. Controlled Experiments

We conduct a few controlled experiments to see the impact of different parameters on the proposed architecture. We elaborate on all these experiments with their results as follows.

#### 4.6.1. Camera Viewpoints

The camera viewpoints (azimuth and elevation angles) of the input 2D pose profoundly impact the proposed retrieval and reconstruction process. We investigate the impact of camera viewpoints for all possible angles—0${}^{\circ }$ to 360${}^{\circ }$ for azimuth angle and 0${}^{\circ }$ to 180${}^{\circ }$ for elevation angle—at which the 2D input pose may be captured. For that purpose, we conduct a few experiments on 100 2D synthetic poses for each action category that we select randomly from the input query 2D dataset ${\mathrm{SDS}}_{1}$.

**Figure 15:**
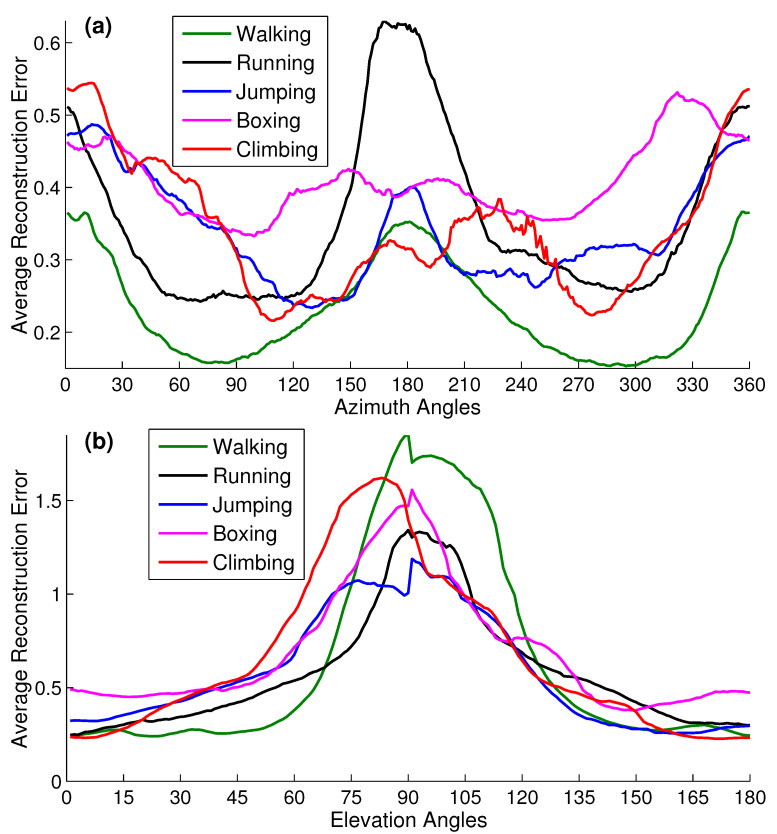
Impact of the camera view angles of the 2D input query pose. The average reconstruction error *recon-err* is computed when the camera view angles of the 2D input query pose are: (**a**) the elevation angle = 30${}^{\circ }$ while the azimuth angle changes from 0–360${}^{\circ }$; (**b**) the azimuth angle = 30${}^{\circ }$ while the elevation angle changes from 0–180${}^{\circ }$. For this experiment, we select 100 synthetic 2D input poses randomly for each action class, taken from the input testing dataset ${\mathrm{SDS}}_{1}$.

In the first scenario, we see the impact of the azimuth angles. We generate 2D synthetic poses for all five action classes by utilizing a weak perspective camera model with elevation angle at 30${}^{\circ }$ and azimuth angles ranging from 0–360${}^{\circ }$. The results reported in Figure 15a reveal that the reconstruction error is minimal at the side-view—the angle between 45–105${}^{\circ }$ and 255–315${}^{\circ }$—for walking, running, and boxing categories of actions while for jumping and climbing actions, the reconstruction error is minimum at 100–150${}^{\circ }$ as well as at 240–270${}^{\circ }$. At the side-view, the walking, running, and boxing action poses are more prominent than any other view. Generally, our approach produces good results for all view directions, either it is a profile view or a frontal view.

In the second scenario of elevation angles, we generate 2D synthetic poses for all action categories through a weak perspective camera model with azimuth angle at 30${}^{\circ }$ and the elevation angles spanning from 0–180${}^{\circ }$. The results presented in Figure 15b demonstrate that the reconstruction error increases at the head-mounted camera views, i.e., at 70${}^{\circ }$ to 110${}^{\circ }$ comparatively. At that specific viewpoints, most of the 2D pose joints overlap with each other, and as a result, it becomes indistinctive, which ultimately leads towards the inappropriate Knn retrieval and finally yields into higher average reconstruction error, *recon-err*.

#### 4.6.2. Joints’ Sensitivity

**Figure 16:**
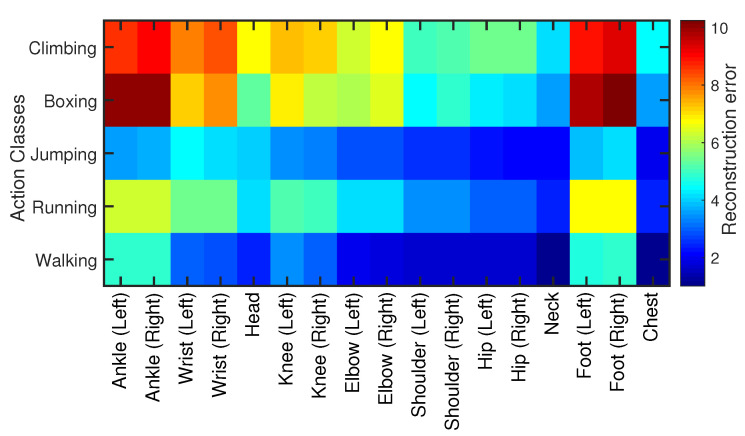
The sensitivity measure for each individual joint involved in the skeleton for all five action classes. We compute Euclidean distance for each joint and is color-coded.

We also check and measure every joint’s sensitivity in our skeleton model except the root joint. For that purpose, we evaluate our proposed methodology joint-wise in terms of reconstruction error for all activity classes. The results reported in Figure 16 illustrate that the end effectors, i.e., wrist joints, ankle joints, and the feet joints, are more sensitive and erroneous for all action categories than all other skeleton joints. It is because these joints are more capable of moving all-around freely. The joints like shoulder joints, neck joints, and hip joints are less sensitive and erroneous as expected since these joints have limited movement capacity. In conclusion, we found that the joints’ sensitivity is directly proportional to the joints’ movement in all directions. That’s why the end effectors seem to be comparatively more sensitive joints.

## 5. Conclusions

This paper proposes a novel and efficient architecture for 3D human pose search and retrieval that leads to 3D human pose estimation from a single static 2D image that is either a synthetic image, an annotated 2D image, an *in-the-wild* real image, or a hand-drawn sketch. We devise a set of feature sets through different coalitions of subsets of skeleton joints for fast search and retrieval of Knn from the MoCap dataset. We evaluate these feature sets based on similarity retrieval, the average reconstruction error, computational time complexity, and memory consumption. We exploit further these retrieved Knn to infer the ultimate 3D human pose. We also benefit from these retrieved Knn to predict the weak perspective camera parameters. For 3D pose reconstruction, we formulate an objective function that consists of multiple energy terms. We evaluate our proposed approach quantitatively on 43,809 synthetic 2D static images and the annotated 2D images from the Human3.6M dataset. For qualitative analysis, we deploy a variety of *in-the-wild* internet real images and 2D hand-drawn human poses. With a pool of experiments conducted on such a large heterogeneous 2D input testing dataset, we have found that our developed system convincingly outperforms other state-of-the-art methods on CMU, HDM05, and Human3.6M MoCap datasets. Our proposed approach achieves better performance than compared approaches even in case of 2D noisy inputs. Moreover, our system is robust enough to yield very plausible 3D reconstruction results with hand-drawn sketches. Our system takes roughly 0.668 s per 2D input pose for retrieval and the final 3D reconstruction.

## Figures and Tables

**Table 1:** The synthetic 2D input testing dataset ${\mathrm{SDS}}_{1}$ created from the CMU motion files using the camera matrix with random camera parameters.

Synthetic 2D Input Testing Dataset (${\mathrm{SDS}}_{1}$).						
**Components**	**Walking**	**Running**	**Jumping**	**Boxing**	**Climbing**	**Total**
No. of human poses	13,509	2970	5913	9128	12,289	43,809
No. of subjects	8	8	4	4	1	25

**Table 2:** Computational efficiency (sec.) for all feature sets, i.e., $F_{5}^{im}$, $F_{7}^{im}$, $F_{9}^{im}$, $F_{11}^{im}$, and $F_{14}^{im}$ concerning time spent on construction of the *knowledge-base*, the *k*d-tree, and on the process of retrieval and reconstruction. This experiment is conducted on 360K CMU dataset poses, and 24×7 number of virtual cameras are deployed. Note that both (**a**) and (**b**) are the pre-processing steps.

Computational Efficiency in s for Feature Sets.					
**Components**	**$F_{5}^{im}$**	**$F_{7}^{im}$**	**$F_{9}^{im}$**	**$F_{11}^{im}$**	**$F_{14}^{im}$**
(**a**) The development of the *knowledge-base*	30.55	42.12	54.78	67.86	77.63
(**b**) The construction of *k*d-tree	97.60	118.12	130.26	144.49	197.73
(**c**) The process of retrieval and reconstruction	0.53	0.56	0.62	0.67	0.96

**Table 3:** A quantitative evaluation of our proposed approach on testing input dataset ${\mathrm{SDS}}_{1}$. (**a**) and (**b**) reports the quantitative evaluation results on MoCap datasets, ${\mathrm{MDS}}_{cmu}$ and ${\bar{\mathrm{MDS}}}_{cmu}$, respectively, while (**c**) shows results when the MoCap dataset is ${\mathrm{MDS}}_{hdm}$. The best results are shown in bold.

Quantitative Evaluation on ${\mathrm{MDS}}_{\mathrm{cmu}}$, ${\bar{\mathrm{MDS}}}_{\mathrm{cmu}}$, and ${\mathrm{MDS}}_{\mathrm{hdm}}$.							
**Methods**	**Error Metrics**	**Walking**	**Running**	**Jumping**	**Boxing**	**Climbing**	**Average**
(**a**) CMU MoCap dataset is used, ${\mathrm{MDS}}_{cmu}$							
PCA (PC-18)	*recon-err*	0.546	0.573	0.454	0.694	0.651	0.583
	*recon-rate*	21.6%	18.0%	22.6%	8.1%	17.1%	17.48%
[35]	*recon-err*	0.446	0.453	0.374	0.584	0.533	0.478
	*recon-rate*	29.6%	23.0%	31.6%	10.7%	20.1%	23.0%
[36]	*recon-err* (λ=0.0)	0.360	0.417	0.343	0.579	0.560	0.452
	*recon-rate* (λ=0.0)	53.4%	29.8%	34.12%	13.3%	21.7%	30.46%
	*recon-err* (λ=0.1)	0.300	0.390	0.322	0.530	0.528	0.414
	*recon-rate* (λ=0.1)	71.2%	35.1%	39.5%	17.0%	27.9%	38.14%
	*recon-err* (λ=0.2)	0.260	0.385	0.316	0.535	0.522	0.404
	*recon-rate* (λ=0.2)	73.9%	38.2%	41.6%	16.4%	27.0%	39.42%
	*recon-err* (λ=0.3)	0.272	0.432	0.321	0.534	0.526	0.417
	*recon-rate* (λ=0.3)	70.4%	34.0%	40.2%	16.8%	28.1%	37.9%
[8]	*recon-err*	0.195	0.286	0.196	0.396	0.409	0.296
	*recon-rate*	84.7%	62.1%	84.5%	45.1%	40.6%	63.4%
Our App.	*recon-err*	**0.183**	**0.253**	**0.179**	**0.365**	**0.391**	**0.274**
	*recon-rate*	**85.8%**	**64.5%**	**85.8%**	**49.2%**	**41.9%**	**65.44%**
(**b**) CMU MoCap dataset is used, ${\bar{\mathrm{MDS}}}_{cmu}$							
Our App.	*recon-err*	0.207	0.331	0.227	0.413	0.529	0.341
	*recon-rate*	82.9%	51.7%	77.8%	39.1%	22.1%	54.72%
(**c**) HDM05 MoCap dataset is used, ${\mathrm{MDS}}_{hdm}$							
[8]	*recon-err*	0.317	0.406	0.237	0.554	0.595	0.422
	*recon-rate*	54.9%	29.3%	85.4%	6.4%	17.6%	38.7%
Our App.	*recon-err*	**0.301**	**0.391**	**0.213**	**0.529**	**0.568**	**0.4**
	*recon-rate*	**56.1%**	**30.9%**	**86.9%**	**8.73%**	**18.9%**	**40.31%**

**Table 4:** The average 3D reconstruction error (mm) on the Human3.6M MoCap dataset with 2D ground-truth for subject S11. The best results are shown in bold.

Quantitative Evaluation on ${\mathrm{MDS}}_{h3.6m}$.																
**Methods**	**Dir.**	**Disc.**	**Eat**	**Greet**	**Ph.**	**Pose**	**Pur.**	**Sit**	**SitD.**	**Smoke**	**Photo**	**Wait**	**Walk**	**WalkD.**	**WalkT.**	**Mean**
[47]	51.9	45.3	62.4	55.7	49.2	56.0	46.4	56.3	76.6	58.8	79.1	58.9	35.6	63.4	46.3	56.1
[10]	60.0	54.7	71.6	67.5	63.8	61.9	55.7	73.9	110.8	78.9	96.9	67.9	47.5	89.3	53.4	70.5
[49]	53.3	46.8	**58.6**	61.2	56.0	58.1	48.9	**55.6**	73.4	60.3	76.1	62.2	35.8	**61.9**	51.1	57.5
[48]	59.1	63.3	70.6	65.1	61.2	73.2	83.7	84.9	**72.7**	84.3	**68.4**	81.9	57.5	75.1	49.6	70.0
Our App.	**50.5**	**42.7**	60.7	**54.9**	**48.1**	**54.1**	**44.8**	55.7	73.6	**57.1**	77.6	**57.3**	**33.5**	62.2	**43.8**	**54.4**

**Table 5:** Quantitative evaluation of the proposed architecture on a noisy input data with different levels of standard deviations σ. The best results are shown in bold.

Quantitative Evaluation on Noisy Input Data.						
**Methods**	**Error Metrics**	σ **(0.0)**	σ **(0.1)**	σ **(0.2)**	σ **(0.3)**	σ **(0.4)**
[36]	*recon-err*	0.414	0.449	0.485	0.561	0.630
	*recon-rate*	32.6%	28.7%	24.4%	18.1%	13.1%
[35]	*recon-err*	0.466	0.497	0.558	0.634	0.704
	*recon-rate*	23.9%	20.5%	13.8%	9.3%	4.8%
Our App.	*recon-err*	**0.271**	**0.326**	**0.431**	**0.524**	**0.617**
	*recon-rate*	**67.3%**	**52.9%**	**38.1%**	**34.9%**	**33.8%**

## Data Availability

Not applicable.
